# Voltammetric measurement of catechol-O-methyltransferase inhibitor tolcapone in the pharmaceutical form on the boron-doped diamond electrode

**DOI:** 10.55730/1300-0527.3650

**Published:** 2023-11-02

**Authors:** Musa KIRAN, Yavuz YARDIM

**Affiliations:** 1 Department of Analytical Chemistry, Faculty of Chemistry, University of Van Yüzüncü Yıl, Van, Turkiye; 2 Department of Analytical Chemistry, Faculty of Pharmacy, University of Van Yüzüncü Yıl, Van, Turkiye

**Keywords:** Tolcapone, boron-doped diamond electrode, voltammetric techniques, pharmaceutical form

## Abstract

This study presents an electroanalytical approach to measure the catechol-O-methyltransferase (COMT) inhibitor tolcapone (TOL) using a boron-doped diamond (BDD) electrode. The application of cyclic voltammetry (CV) technique revealed that TOL exhibited a distinct, diffusion-controlled, irreversible anodic peak at a potential of approximately +0.71 V (vs. Ag/AgCl) in a 0.1 mol L^−1^ phosphate buffer solution (PBS) with a pH of 2.5. The oxidation of TOL is highly dependent on the pH and supporting electrolytes. Based on the data obtained from the pH investigation, a proposed mechanism for the electro-oxidation of TOL is suggested. Using the square wave voltammetry (SWV) technique, a satisfactory linear relationship was observed at approximately +0.66 V in a 0.1 mol L^−1^ PBS with a pH of 2.5. The presented method exhibited linearity within the concentration range between 1.0–50.0 μg mL^−1^ (3.7 × 10^−6^–1.8 × 10^−4^ mol L^−1^), with a limit of detection (LOD) of 0.29 μg mL^−1^ (1.1 × 10^−6^ mol L^−1^). The BDD electrode demonstrated good selectivity against inorganic ions and filler materials interference. Finally, the suitability of the developed approach was assessed by measuring TOL in tablet formulations, resulting in favorable recoveries ranging from 103.4% to 106.2%.

## 1. Introduction

Parkinson’s disease (PD) is a prevalent neurodegenerative disorder, ranking as the second most common after Alzheimer’s disease (AD). It is estimated to affect around 1%–2% of people aged 65 years and older [1]. The existing therapeutic approaches for PD aim to temporarily alleviate symptoms by restoring the loss of dopamine (DOP) through “dopamine replacement therapy.” Levodopa (L-Dopa), a direct precursor of DOP, and other drugs that slow down DOP metabolism are used to achieve this goal [2,3]. However, after receiving L-Dopa treatment for 5–7 years, many PD patients experience dysmotility and reactive movement fluctuations. As a result, there has been an increased research interest in maintaining plasma L-Dopa concentration, prolonging its half-life, and developing drugs with a higher striatum DOP concentration [4–6]. Catechol-O-methyltransferase (COMT) inhibitors are crucial medications for managing PD. The initial generation of COMT inhibitors was excessively toxic to be approved for use, but second-generation COMT inhibitors, including tolcapone (TOL), have been found to be effective in providing symptomatic relief for certain Parkinson’s patients with an acceptable toxicity profile. TOL has been shown to predominantly inhibit COMT in the peripheral nervous system, with less impact on the central nervous system [7–10]. In the existing literature, the number of analytical methods for quantifying TOL is limited. Therefore, it is of utmost importance to strive towards the development of reliable and efficient analytical approaches to provide quality of commercially accessible medications. Typically, chromatographic techniques have been employed for the determination of TOL in tissue samples and pharmaceutical formulations. High-performance liquid chromatography (HPLC) [11–13], and HPLC tandem mass spectrometry (HPLC-MS/MS) have been reported for the sensing of TOL. On the other hand, only one voltammetric study with mathematical modeling of TOL, along with other antiparkinsonian agents, was found in the literature [14]. In the study, the electrochemical properties of TOL and other antiparkinsonian agents on the gold disc electrode were investigated via differential pulse voltammetry (DPV) and cyclic voltammetry (CV), and a method for multiple determinations with mathematical modeling was proposed. Among electroanalytical methods, the voltammetry technique presents promising prospects due to its fast analysis, simplicity, affordability of equipment, and utilisation of low-toxicity chemicals such as buffer solutions. Additionally, the choice of the working electrode plays a role in achieving satisfactory selectivity and acceptable sensitivity. Voltammetry offers an advantage over nonelectrochemical methods by allowing the identification of the complex oxidation-reduction behavior of analytes using electroactive agents. This knowledge is essential for the development of novel approaches to evaluate the therapeutic and toxic effects of relevant substances. Widespread studies conducted over the past three decades have highlighted the increasing interest in boron-doped diamond (BDD) is an environmentally friendly electrode material that belongs to a relatively recent category of carbon-based materials. BDD has shown potential applications in various fields, including environmental investigations, analytical chemistry, biology, materials science, and other domains [15–17].

The BDD electrode provides the widest potential window among any electrode substances, including conventional and sp2 carbon materials (carbon paste, pyrolytic graphite, glassy carbon, and others), as well as various metals (platinum gold). Other differentiating characteristics are its strong chemical and physical sturdiness, which provides the repeatability of the signal, low and stable background currents, including low adsorption of most contaminants (due to the formation of diamond containing sp3 hybridized carbon atoms), and especially low sensitivity to dissolved oxygen. As a result, the unique properties and characteristics of the BDD electrode make it suitable for various electroanalytical applications [18–21].

As previously mentioned, a literature review indicated that chromatographic methods were commonly employed for the quantitative analysis of TOL. However, these techniques typically involve the use of hazardous organic solvents in large quantities, expensive instrumentation, and time-consuming pretreatment. In contrast, the current approach using the SWS voltammetry technique offers several desirable characteristics, including rapid and simple sample treatment, minimal use of organic reagents, acceptable repeatability, and sufficient sensitivity for real sample applications [17,22,23].

The primary objective of this study is to establish a precise, simple, and rapid electroanalytical method for the detection of TOL in an acidic medium, employing the BDD electrode. The developed method was successfully applied to commercialized tablet samples by utilizing the optimum experimental conditions.

## 2. Experimental

### 2.1. Chemicals

ChemScene LLC (USA) was the supplier of the TOL standard reference (purity: 99.91%). The high-purity water obtained from the Milli-Q water purification system (Millipore, resistivity ≥ 18.2 MΩ) was utilized to prepare analytical-grade reagents, including H_2_SO_4_ (0.1 mol L^−1^), HNO_3_ (0.1 mol L^−1^), HClO_4_ (0.1 mol L^−1^), acetate buffer solution (ABS, 0.1 mol L^−1^, pH 4.7), phosphate buffer solution (PBS, 0.1 mol L^−1^, pH 2.5), and Britton-Robinson buffer (BR, 0.04 mol L^−1^, pH 2.0–8.0). The stock solution of TOL (1.0 mg mL^−1^) was dissolved in ethanol, and the resulting solutions were diluted in the chosen electrolytes.

### 2.2. Instruments and operating procedures

Electrochemical measurements were performed using a μAutolab type III electrochemical analyzer from Metrohm Autolab B.V., the Netherlands. The data obtained were analyzed using GPES software (Version 4.9). To enhance the accuracy of the square wave voltammograms, baseline correction and smoothing were performed using the Savitzky-Golay algorithm with a peak width of 0.01 V. The electrochemical measurements were conducted at room temperature using a three-electrode glass cell system with a volume of 10 mL. The system included a platinum wire auxiliary electrode, a BDD working electrode (with a diameter of 3 mm and a boron content of 1000 ppm, Windsor Scientific Ltd., UK), and an Ag/AgCl reference electrode (3 mol L^−1^ NaCl, model RE-1, BAS, USA). The surface of the BDD electrode was analyzed via scanning electron microscopy (SEM) using a Zeiss Sigma 300 microscope, along with energy-dispersive X-ray (EDX) spectroscopy and an In-Lens (SE1) detector at an accelerating voltage of 10 kV. A pH meter used for pH measurements was a WTWinoLab pH 720 m system (Xylem, New York, USA).

The working (BDD) electrode was pretreated to minimize the effect of TOL signals in the voltammetric cell. Before each experimental day, the BDD electrode was subjected to a cathodic voltage of −1.8 V for 180 s in a 0.5 mol L^−1^ H_2_SO_4_ solution. Before each individual measurement, the electrode was pretreated with −1.8 V for 60 s in a 0.5 mol L^−1^ H_2_SO_4_ solution. This procedure ensures a clean electrode surface and prevents the adsorption of TOL oxidation compounds.

The reaction mechanism and electrochemical behavior of TOL on the BDD electrode in the chosen supporting electrolyte were studied utilizing the cyclic voltammetry (CV) technique. Subsequently, square wave voltammetry (SWV) was employed to identify the optimal conditions, such as the supporting electrolyte at different pH values and SWV parameters, to enhance the electrode’s sensitivity for TOL analysis. Additionally, the developed approach was evaluated for its analytical performance, including the effect of interfering agents, and its application.

To perform TOL sensing using SWV, a three-electrode configuration was placed in a voltammetric cell including TOL and a 0.1 mol L^−1^ PBS at pH 2.5. Anodic scanning was conducted within the potential range of 0 to +1.1 V. Carried out using the standard addition method, the analysis of TOL in tablet samples was performed, and all electrochemical measurements were conducted in triplicate.

### 2.3. Sample preparation

A tablet sample of TOL, containing 100 mg of the compound (Tasmar Meda Pharma Co.), was obtained from a pharmacy. The tablets were pulverized using a mortar and pestle. An appropriate amount of the obtained powder, equal to 25.0 mg of TOL, was added to a 25 mL calibrated bottle and ethanol was added to fill the volume. The mixture was stirred continuously for around 15 min until all components of the mixture were dissolved. Using a micropipette, 50 μL of the solution was transferred into the voltammetric cell (contains 0.1 mol L^−1^ PBS at pH 2.5), and analyzed after adding 1.0, 2.5, 5.0, 10, and 20 μg mL^−1^ TOL to the sample, the TOL amount in the sample was detected via utilising a method of standard addition.

## 3. Results and discussion

**Figure 1:**
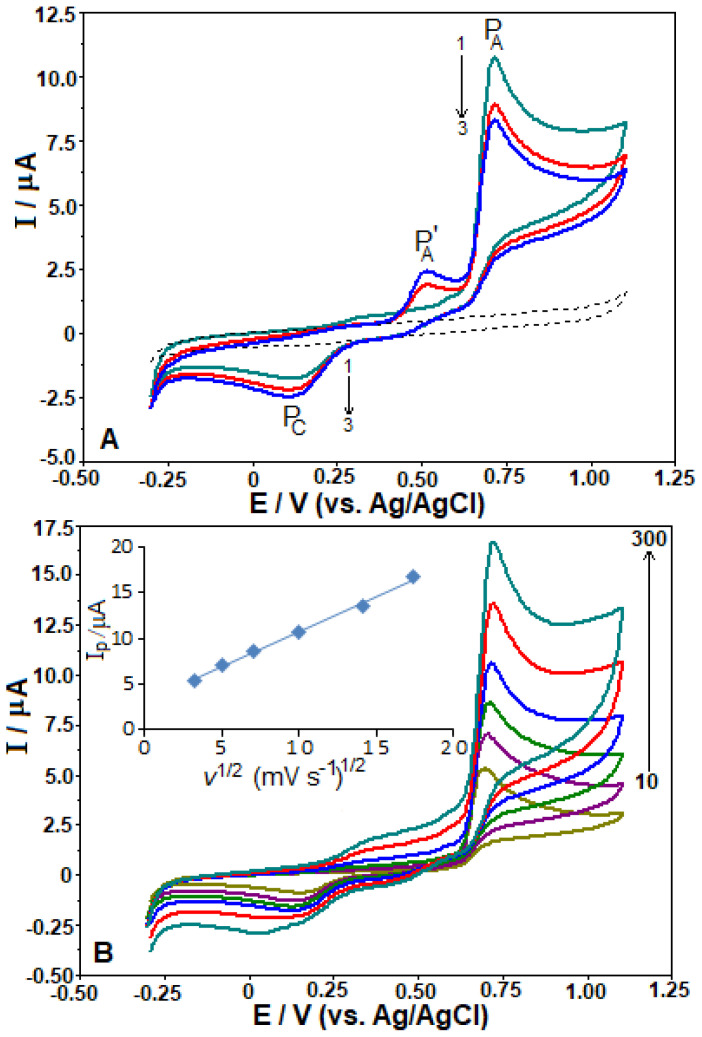
Repeated cyclic voltammograms (CVs) were obtained at a scan rate of 100 mV s^−1^ for 150 μg mL^−1^ TOL (A), CVs conducted at several scan rates (10, 25, 50, 100, 200, and 300 mV s^−1^) for 150 μg mL^−1^ TOL (B) in 0.1 M PBS at pH 2.5 on the BDD electrode. A: Dashed lines were used to represent the background current. B: Linear dependences of *i*_p_ versus *v* ^1/2^ were displayed in the inset.

Using the CV technique with the BDD electrode, the electrochemical properties of TOL were evaluated. Three sequential CVs were recorded for 150 μg mL^−1^ of TOL using a potential scanning rate of 100 mV s^−1^. The measurements were performed in a solution of 0.1 mol L^−1^ PBS at pH 2.5. TOL exhibited a well-defined oxidation peak at a potential (P_A_) of approximately +0.76 V (Figure 1A). In the opposite scan, a reduction peak (P_C_) was recorded at around +0.12 V, accompanied by a small additional anodic peak (P_A׀_) (at about 0.50 V) at less positive potential than the P_A_, during the second and subsequent scans. With an increase in the number of scans, the peak height of P_A_ decreased gradually, while the intensity of P_C_/P_A׀_ slightly increased. These findings suggest that the oxidation peak at P_A_ is not reversible, while the pair of redox peaks at P_C_/P_A׀_ is indicative of a redox process. This could be due to the formation of byproducts during the main electrooxidation step. As a result, while sequential CVs were recorded, the main oxidation peak (P_A_) was found to decrease, possibly due to the accumulation of TOL and/or its oxidation products on the BDD electrode, leading to deactivation or fouling. The influence of potential scan rate (*ν*) on the anodic peak current of TOL (150 μg mL^−1^) was examined using the CV technique in a 0.1 mol L^−1^ PBS solution at pH 2.5. The scan rate range was set from 10 to 300 mV s^−1^ (Figure 1B) to examine the kinetics of TOL oxidation on the BDD electrode. As the square root of the scan rate (*ν*^1/2^) increased, the anodic peak currents of TOL also increased. The relationship between TOL’s oxidation peak currents and *ν*^1/2^ was evaluated using the equation: *i*_pa_ (μA) = 0.772 *ν*^1/2^ (mV s^−1^) + 3.023, with a correlation coefficient (*r*) of 0.9983 (*n* = 6). Additionally, a linear dependence was observed when plotting log *i*_p_ against log *v*, following the equation: log *i*_p_ (μA) = 0.326 log *v* (mV s^−1^) + 0.392, with a *r* of 0.9964. Close to the theoretically anticipated values of 0.5 for diffusion-controlled processes is the obtained slope (~0.39). Hence, it might be considered that the TOL oxidation is controlled via a diffusion.

**Figure 2:**
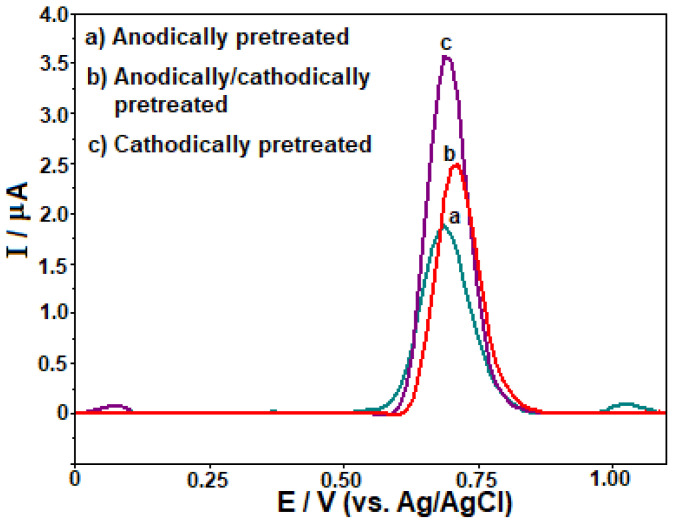
SW voltammograms were recorded for 50.0 μg mL^−1^ TOL in 0.1 M PBS at pH 2.5 on the BDD electrode after applying different electrochemical pretreatments. SWV parameters: Δ*E*_s_ = 10 mV; Δ*E*_sw_ = 40 mV; *f* = 50 Hz.

**Figure 3:**
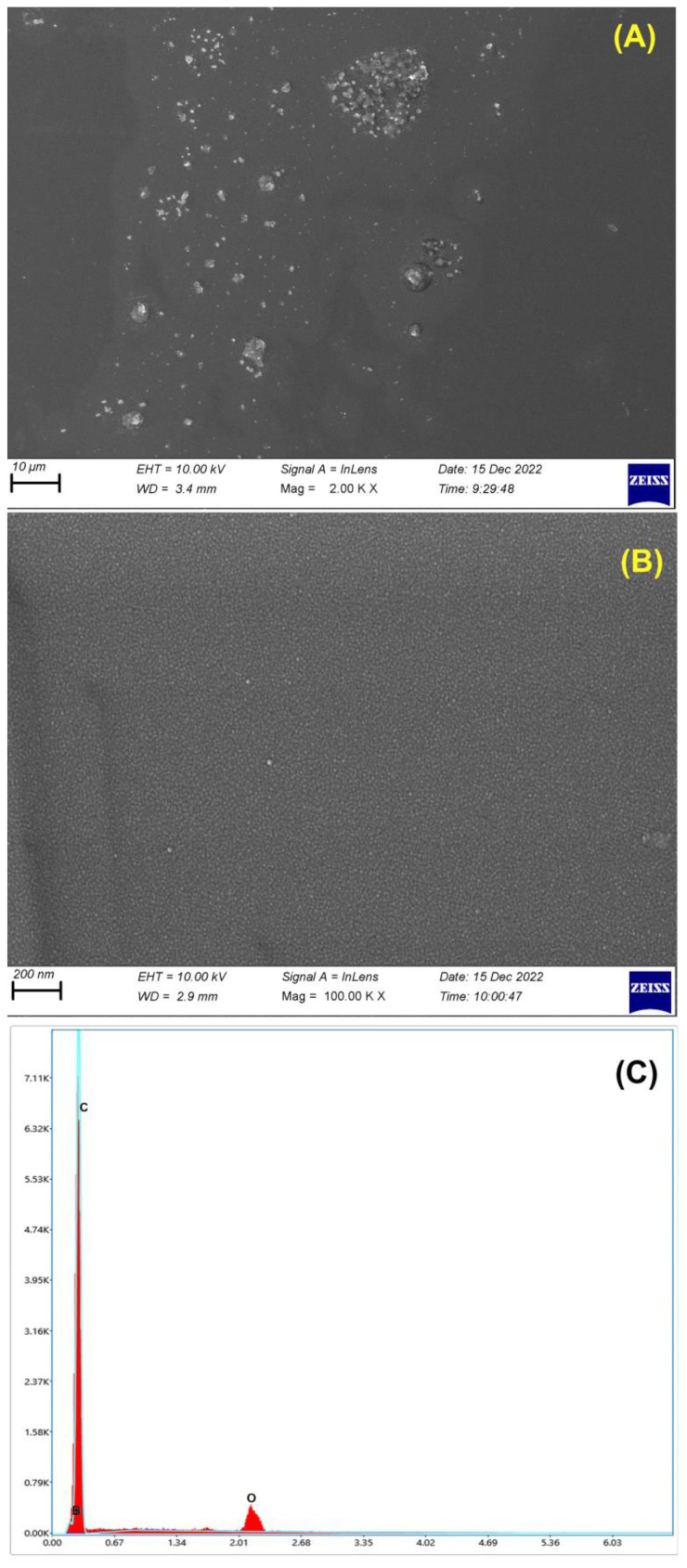
SEM images in different scale (10 μm and 0.2 μm) for the BDD electrode (A–B). SEM-EDX spectrum and elemental composition of the BDD electrode (C).

In the subsequent stage of the study, the square wave (SW) mode was chosen for its lower consumption of electroactive agents, improved sensitivity, and rapid analysis rate. Preliminary examinations indicated that the unmodified BDD electrode faced challenges related to passivation, particularly at high TOL concentrations. Consequently, the untreated BDD electrode yielded unsatisfactory results in terms of repeatability and sensitivity. SWV technique was employed to assess the performance of three distinct methods in handling and evaluating the response of the BDD electrode towards 50 μg mL^−1^ TOL in a 0.1 mol L^−1^ PBS solution at pH 2.5. Initially, the BDD electrode was subjected to anodic pretreatment (APT) by applying a potential of +1.8 V for 180 s in 0.5 mol L^−1^ H_2_SO_4_. Subsequently, the effect of cathodic pretreatment (CPT) on the BDD electrode was examined by applying a voltage of −1.8 V for 180 s in 0.5 mol L^−1^ H_2_SO_4_. Eventually, a combination of anodic and cathodic pretreatment was carried out on the BDD electrode. The CPT procedure was conducted on the BDD electrode, as it yielded the most sensitive results for TOL analysis in this study (Figure 2). The CPT technique might have speeded up of electron transfer of TOL on the BDD electrode compared to the others. As a result, this procedure was subsequently employed in the subsequent stages of the work. Hereinafter it was indicated CPT-BDD electrode. The analyses of SEM and SEM-EDX were carried out to examine the BDD electrode surface morphology. The SEM images presented in Figure 3A and 3B indicate that the surface of the BDD electrode was partially rough under certain working conditions, but overall, it possesses a favorable morphological structure. Additionally, the BDD electrode’s elemental composition was confirmed by analyzing the spectrum of SEM-EDX that is presented in Figure 3C.

**Figure 4:**
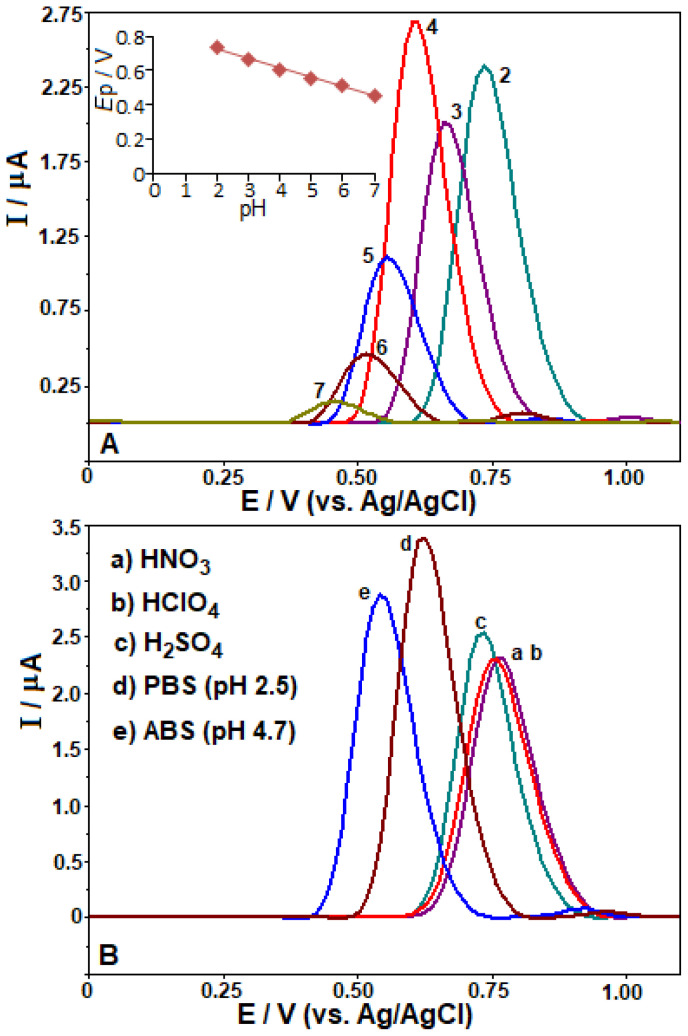
SW voltammograms for 50 μg mL^−1^ TOL in BR buffer (pH 2.0–7.0) (A) as well as various electrolytes at various pH ranges (B) at the CPT-BDD electrode. In Figure 2, the other working conditions are shown.

SWV technique was employed to examine the influence of different pH values on the oxidation signal responses of TOL on the BDD electrode in order to determine the optimal medium. SWV measurements were conducted in BR buffer (pH range of 2.0–7.0) for a 50 μg mL^−1^ TOL, and the obtained results are demonstrated in Figure 4A. The data presented in Figure 4A indicate that as the pH value increased in the range of pH 2.0–7.0, there was a shift towards lower positive values in the anodic peak potentials. It should be underlined that no signal was detected at pH values greater than 7.0 under the working conditions. The correlation between the anodic potential of TOL on the BDD electrode and pH (from 2.0 to 7.0) can be described by the equation *E*p (V) = −0.053 pH + 0.827 (*r* = 0.9942), indicating that the oxidation of TOL on the electrode is influenced by the pH of the solution. The obtained results demonstrate that the electrode reaction exhibits a stoichiometry involving an equal number of electrons and protons, as evidenced by the slopes of 0.053 V/pH, which closely approximate the theoretical value of 0.059 V. Based on the results obtained, and taking into consideration a previous report that investigated the electrochemical oxidation of entacapone, which has a structure similar to TOL [24], it is possible to briefly explain the oxidation mechanism of TOL. TOL is composed of a dihydroxybenzene group, which undergoes oxidation of the hydroxyl groups (OH groups), leading to the formation of a phenoxy radical. This radical can be further oxidized to a quinone, as previously reported [25, 26]. Considering the information presented above and the obtained results, it can be hypothesized that the redox process involves the oxidation of TOL to o-quinone, which is the reaction involving two electrons and two protons. A potential oxidation mechanism of TOL on the CPT-BDD electrode is illustrated in Scheme. In the different electrolytes, the SWV signals are presented in Figure 4B. By using 0.1 mol L^−1^ H_2_SO_4_, HNO_3_, HCIO_4_, PBS pH 2.5, and ABS pH 4.7, anodic peak potentials of +0.73, 0.76, 0.76, 0.63, and 0.54 V were recorded, respectively, with the oxidation peak currents of 2.54, 2.32, 2.31, 3.39 and 2.88 μA, respectively. As observed from Figure 3A and B, the best SWV signal was obtained at pH 2.5 (PBS) on the CPT-BDD electrode for 50 μg mL^−1^ TOL. Performed was a comparison between two pulse techniques, SWV and differential pulse voltammetry (DPV), to determine the more sensitive technique for measuring the oxidation peak currents of TOL. The findings revealed that SWV exhibited higher sensitivity compared to DPV under identical conditions (see supplementary Figure S1). The findings of the study were similar to a previous study performed on a BDD electrode, which was reported in the work [17]. Hence, further investigations will be carried out utilizing the SWV method.

Ultimately, optimization of SWV parameters including frequency (*f* = 25–125 Hz), step potential (Δ*E*_s_ = 8–18 mV), and pulse amplitude (Δ*E*_sw_ = 30–80 mV) were done for set-up for TOL sensing under the obtained conditions. The optimization was carried out by maintaining two parameters constant while varying one parameter at a time. The best parameters obtained at the end of the experiments were; *f* = 100 Hz, Δ*E*_s_ = 12 mV, and Δ*E*_sw_ = 60 mV.

**Figure 5:**
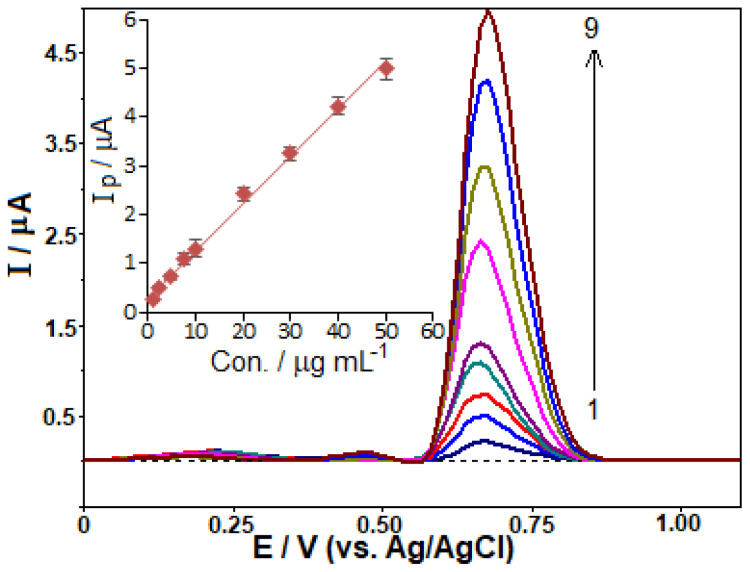
SW voltammograms with TOL concentrations of (1–9) 1.0, 2.5, 5.0, 7.5, 10.0, 20.0, 30.0, 40.0, and 50.0 μg mL^−1^ in 0.1 M PBS at pH 2.5 on the CPT-BDD electrode. The calibration graph for TOL measurement is presented inset. SWV parameters: Δ*E*_s_ = 12 mV; Δ*E*_sw_ = 60 mV; *f* = 100 Hz.

After fine-tuning the experimental conditions, the performance of the analysis was assessed by measuring the oxidation peak currents with respect to different concentrations of TOL. The recorded and acquired results were demonstrated in Figure 5, and Table 1 presents the acquired analytical parameters. The LOD and LOQ values were determined to be 0.29 μg mL^−1^ (1.1 × 10^−6^ mol L^−1^) and 0.97 μg mL^−1^ (3.6 × 10^−6^ mol L^−1^), respectively, using the 3 *s*/*m* and 10 *s*/*m* formula, respectively. The smallest concentration in the calibration range was measured ten times to obtain the standard deviation (*s*), which was then divided by the slope (*m*) of the analytical curve.

The comparison of some published reports with the proposed method is shown in Table 2. Compared to other developed methods such as RP-HPLC [12] and HPLC-MS/MS [13], the proposed technique using CPT-BDD electrode is advantageous in terms of economy, speed, and simpleness, although it may not be as sensitive as the HPLC-MS/MS method.

To assess the precision (Table 1) of the developed approach, the intra-day repeatability (ten repeats) and inter-day repeatability (three consecutive days) for 1.0 μg mL^−1^ of TOL were investigated under the obtained optimum conditions. The obtained values of RSD show that the CPT-BDD electrode is well-suited as a working electrode for the reliable detection of TOL in real samples.

It is important to highlight that the existence of electroactive substances can interfere with the detection of the target agent peak in biological samples or pharmaceutical formulations. To evaluate the selectivity of TOL determination on the CPT-BDD electrode, various molecules, and ions commonly found in urine samples or drug formulations were introduced at concentration ratios of 1:1, 1:10, and 1:50 (TOL: interfering compound) while recording changes in the TOL signal at a concentration of 5.0 μg mL^−1^ under optimal conditions. To determine the limit of tolerance for the anodic signal of TOL, the concentration was adjusted to produce an average error of ±5%. The impact of different interfering substances on the TOL oxidation signal was recorded and summarized in Table 3. Results showed that a 50-fold increase in concentrations of sugars (maltose, glucose, lactose, sucrose), inorganic ions (K^+^, Na^+^, Ca^2+^, Mg^2+^, PO_4_^3−^, etc.), and filler materials (cellulose, magnesium stearate, starch and sodium dodecyl sulfate) did not meaningfully impact the anodic peak current of TOL. In addition, assessing the ability to detect TOL in biological fluids in the existence of uric acid (UA), ascorbic acid (AA), and dopamine (DOP) may have implications for future bioavailability works. The impact of these compounds on the anodic signals of TOL was examined by using different concentration ratios of interfering compounds such as UA, AA, and DOP including 1:1, 1:5, and 1:10. The obtained results revealed that the existence of AA and DOP at the same concentrations did not have a meaningful effect on the anodic peak of TOL. However, the peak currents generated from the oxidation of TOL were observed to be significantly affected by the presence of UA at a 1:1 (TOL:UA) ratio (see supplementary Figure S2). Consequently, this method may have limitations for clinical analysis, particularly for controlling TOL at high concentrations of these agents.

**Figure 6:**
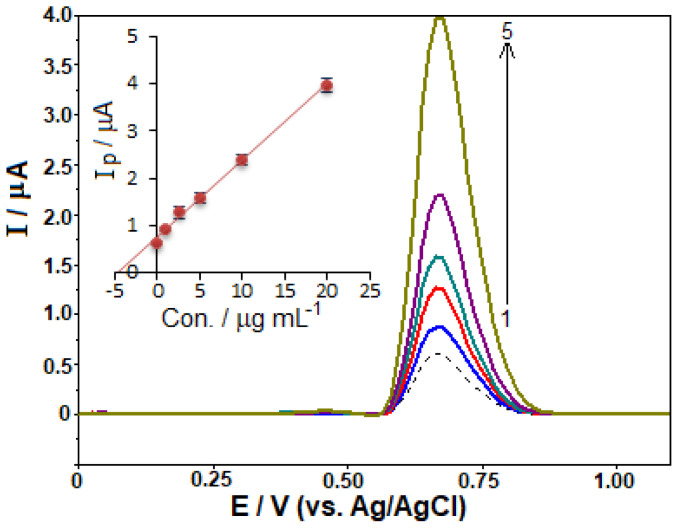
SW voltammograms of the drug sample; dashed line indicating the diluted drug sample, (1–5) after standard additions of 1.0, 2.5, 5.0, 10.0, and 20.0 μg mL^−1^ TOL. The inset displays the result of the analysis using the standard addition method. SWV parameters: Δ*E*_s_ = 12 mV; Δ*E*_sw_ = 60 mV; *f* = 100 Hz.

The developed method was applied to determine the quantity of TOL in a commercially available pharmaceutical formulation, aiming to evaluate its practical applicability. In the experimental section (Section 2.3), the sample preparation procedure and evaluation method were described. The tablet samples were directly analyzed without employing any extraction, filtration, or evaporation steps. The SWVs of the sample and standards added to the sample were graphically evaluated and are shown in Figure 6 [ip (μA) = 0.161 C (μg mL^−1^) + 0.756 (*r* = 0.9982)]. Accounting for the successive dilutions of the sample, the amount of TOL in the tablet was determined to be 93.8 mg (RSD of 3.4%), which is close to the label value of 100.0 mg declared by the producer. To validate the presented method, a recovery examination was carried out by transferring appropriate volumes of the standard solution of TOL (with ultimate concentrations of 1.0, 5.0, and 20.0 μg mL^−1^) to a previously examined sample solution in a voltammetric cell. Table 4 shows the corresponding RSD values obtained from three repeated measurements. The good recovery values suggest no significant matrix effect in the tablet form samples.

## 4. Conclusions

As indicated in the introduction, only one previous study in the literature was found that relates to the electrochemical evaluation of TOL. However, the previous study did not provide a detailed electrochemical analysis or voltammetric determination of TOL; instead, mathematical modeling was used. This investigation demonstrates the practicality of using an unmodified BDD electrode in combination with SWV as a cost-effective, simple, rapid, and potentially convenient electrochemical platform. The successful application of this approach in commercial pharmaceuticals without any interference highlights its usefulness. The absence of current studies on the oxidative behavior of TOL suggests that the findings of this work could be valuable for future electroanalytical investigations, both in voltammetry and chromatography, for amperometric target identification using BDD or other electrode substances.

Figure S1 DP (a) and SW (b) voltammograms of 20 μg mL^−1^ TOL in 0.1 M PBS at pH 2.5 the CPT-BDD electrode. DPV parameters: modulation amplitude, 50 mV; step potential, 8 mV and modulation time 0.05 s. SWV parameters: frequency, 50 Hz; step potential, 10 mV; pulse amplitude, 40 mV.

Figure S2 SW voltammograms of TOL (5.0 μg mL^−1^) mixture in the existence of (A) equimolar concentration, 5 and 10-fold excess AA, (B) equimolar concentration, 5 and 10-fold excess DOP and (C) equimolar concentration, 5 and 10-fold excess UA. SWV parameters: frequency, 100 Hz; step potential, 12 mV; pulse amplitude, 60 mV.

## Figures and Tables

**Scheme:**
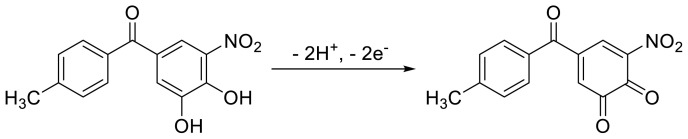
Possible mechanism of TOL oxidation.

**Table 1:** The analytical parameters achieved for the TOL oxidation peak using SWV on the CPT-BDD electrode.

Analytical parameter	P_A_
*E*p	+0.66 V
LWR	1.0–50.0 μgmL^−1^ (3.7 × 10^−6^ – 1.8 × 10^−4^ M)
LRE	*i*_p_ (μA) = 0.097 *C* (μg mL^−1^) + 0.298
*r*	0.9984
LOD	0.29 μg mL^−1^ (1.1 × 10^−6^ M)
LOQ	0.97 μg mL^−1^ (3.6 × 10^−6^ M)
Intra-day repeatability (RSD%, *n* = 10)	5.8
Inter-day repeatability (RSD%, *n* = 3)	7.1

*E*p = peak potential; LWR=linear working range; LRE = linear regression equation; *r* = correlation coefficient; LOD = limit of detection; LOQ, limit of quantification

**Table 2:** The methods were compared in terms of their linear range and limit of quantitation for TOL.

Methods	Linear range (μg mL^−1^)	LOQ (μg mL^−1^)	Ref.

HPLC	0.5–20.0	0.36	[11]
RP-HPLC	5.0–15.0	3.79	[12]
HPLC-MS/MS	0.01–0.5	0.01	[13]
SWV	1.0–50.0	0.97	This work

HPLC; high-performance liquid chromatography, RP-HPLC; reverse phase-HPLC, HPLC-MS/MS; high-performance liquid chromatography-tandem mass spectrometry, SWV; Square wave voltammetry

**Table 3:** The current response of the TOL oxidation peak in the presence of potential interferences.

Interference	Concentration ratios (TOL: interference)	The current change (%)
The inorganic ions	1:50	−7 ± 0.3
The sugars	1:50	−7 ± 0.5
Cellulose	1:50	−5 ± 0.3
Magnesium stearate	1:50	−7 ± 0.4
Starch	1:50	−6 ± 0.4
SDS	1:50	−3 ± 0.1
Ascorbic acid	1:5	+31 ± 1.4
Dopamine	1:10	−37 ± 1.7
Uric acid	1:1	+188 ± 6.8

Inorganic ions; K^+^, Na^+^, Mg^2+^, Ca^2+^, Fe^3+^, Ti^4+^, NO_3_^−^, PO_4_^3−^, The sugars; maltose, lactose, glucose, sucrose, SDS; Sodium dodecyl sulfate.

**Table 4:** The performance of the developed procedure by determining the recovery values of pharmaceutical form samples spiked with TOL standard solutions.

Added^a^ (μg mL^−1^)	Expected^a^ (μg mL^−1^)	Found^a^, ^b^ (μg mL^−1^)	Recovery (%) ± RSD (%)
0	–	4.7	− ± 3.3
1.0	5.7	5.9	103.5 ± 3.2
5.0	9.7	10.3	106.2 ± 2.7
20.0	24.7	25.5	103.2 ± 2.2

a Concentration in the measured solution

b Average of three replicate measurements
