# Supplementary figures and images for: Voltammetric measurement of catechol-O-methyltransferase inhibitor tolcapone in the pharmaceutical form on the boron-doped diamond electrode
Source: Turk J Chem. 2023 Nov 2;48(1):184–94. doi: 10.55730/1300-0527.3650 (PMC10965188; doi:10.55730/1300-0527.3650)

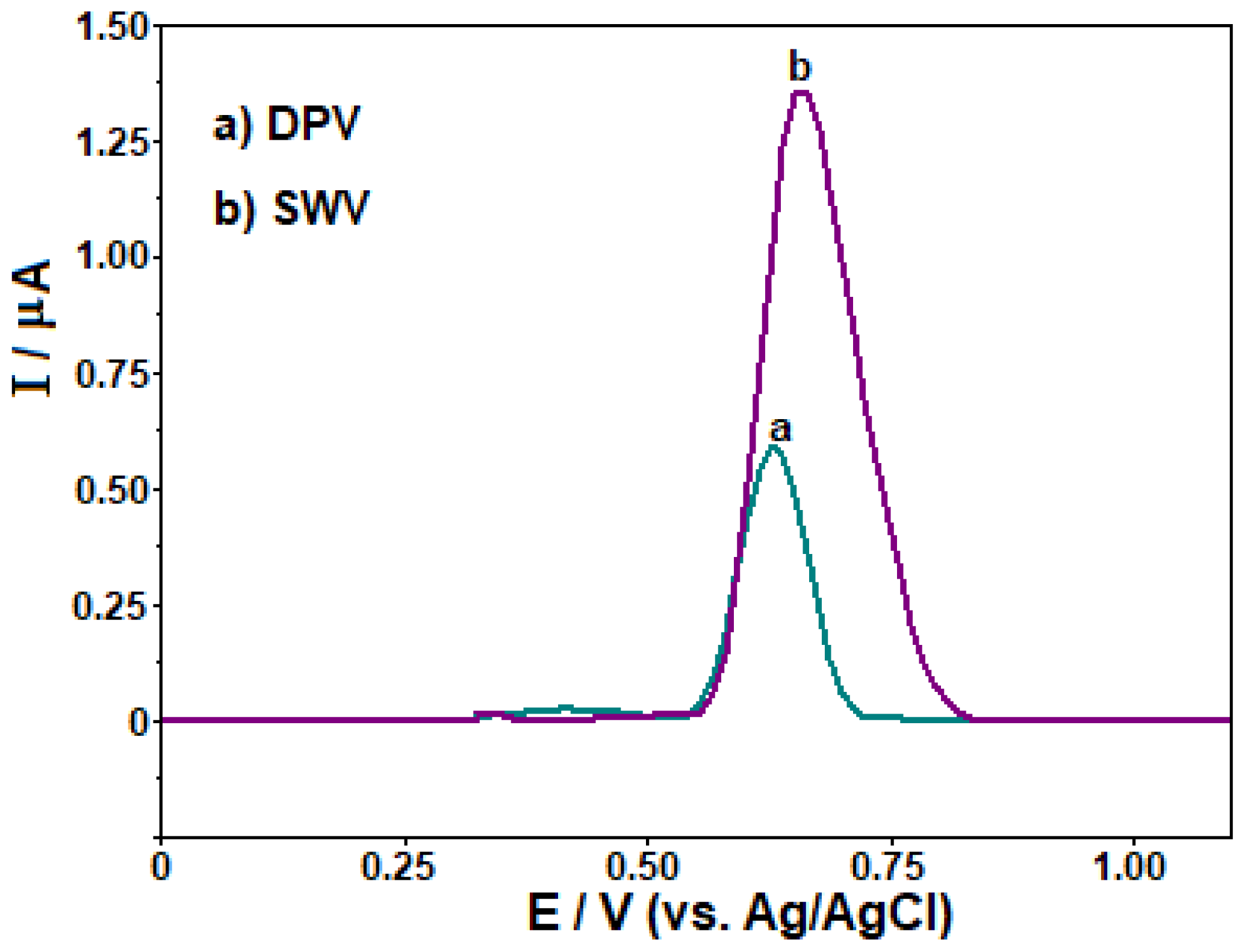

Supplement: Figure S1 — DP (a) and SW (b) voltammograms of 20 μg mL−1 TOL in 0.1 M PBS at pH 2.5 the CPT-BDD electrode. DPV parameters: modulation amplitude, 50 mV; step potential, 8 mV and modulation time 0.05 s. SWV parameters: frequency, 50 Hz; step potential, 10 mV; pulse amplitude, 40 mV. [file tjc-48-01-0184s1.tif]

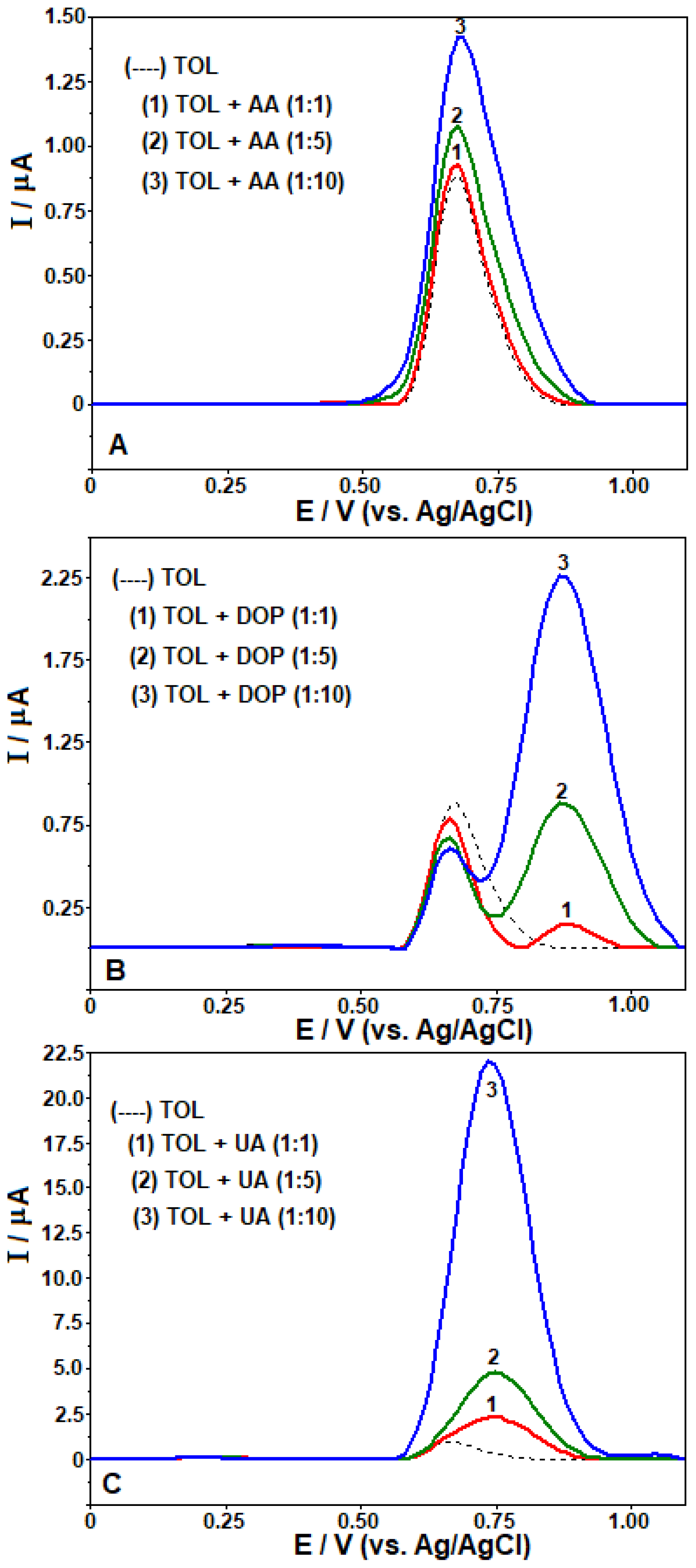

Supplement: Figure S2 — SW voltammograms of TOL (5.0 μg mL−1) mixture in the existence of (A) equimolar concentration, 5 and 10-fold excess AA, (B) equimolar concentration, 5 and 10-fold excess DOP and (C) equimolar concentration, 5 and 10-fold excess UA. SWV parameters: frequency, 100 Hz; step potential, 12 mV; pulse amplitude, 60 mV. [file tjc-48-01-0184s2.tif]
